# Atmospheric-pressure atomic layer deposition: recent applications and new emerging applications in high-porosity/3D materials

**DOI:** 10.1039/d3dt01204b

**Published:** 2023-06-20

**Authors:** M. Chen, M. P. Nijboer, A. Y. Kovalgin, A. Nijmeijer, F. Roozeboom, M. W. J. Luiten-Olieman

**Affiliations:** a Inorganic Membranes, Department of Science and Technology and MESA+ Institute for Nanotechnology, University of Twente PO Box 217 7500 AE Enschede The Netherlands m.chen@utwente.nl m.w.j.luiten@utwente.nl; b Integrated Devices and Systems, Faculty of Electrical Engineering, Mathematics and Computer Science, MESA+ Institute for Nanotechnology, University of Twente PO Box 217 7500 AE Enschede The Netherlands

## Abstract

Atomic layer deposition (ALD) is a widely recognized technique for depositing ultrathin conformal films with excellent thickness control at Ångström or (sub)monolayer level. Atmospheric-pressure ALD is an upcoming ALD process with a potentially lower ownership cost of the reactor. In this review, we provide a comprehensive overview of the recent applications and development of ALD approaches emphasizing those based on operation at atmospheric pressure. Each application determines its own specific reactor design. Spatial ALD (s-ALD) has been recently introduced for the commercial production of large-area 2D displays, the surface passivation and encapsulation of solar cells and organic light-emitting diode (OLED) displays. Atmospheric temporal ALD (t-ALD) has opened up new emerging applications such as high-porosity particle coatings, functionalization of capillary columns for gas chromatography, and membrane modification in water treatment and gas purification. The challenges and opportunities for highly conformal coating on porous substrates by atmospheric ALD have been identified. We discuss in particular the pros and cons of both s-ALD and t-ALD in combination with their reactor designs in relation to the coating of 3D and high-porosity materials.

## Abstract 2

Atmospheric-pressure atomic layer deposition (ALD) is an upcoming technology poised to revolutionize the commercial production of large-area substrates and unlock new applications in high-porosity/3D materials.

## Graphical abstract

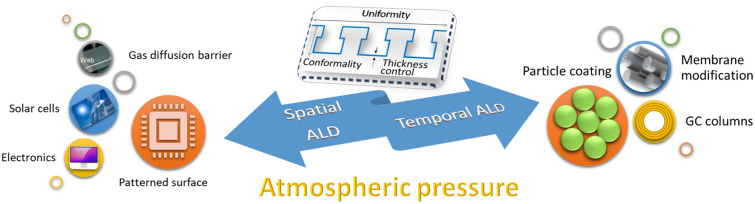

## 1. Introduction

### 1.1. ALD basics and characteristics

Amongst all thin-film deposition techniques, Atomic Layer Deposition (ALD) has become by far the most superior and cost-effective option in realizing thin films with properties and critical dimensions of single-digit nanometer values in complex 3D device architectures requiring extreme edge placement accuracy, layer conformality and shape fidelity.^1^ Based on the self-limiting growth mechanism, ALD allows for the deposition of a wide variety of thin-film materials from the vapor phase. In a typical ALD process, a precursor and its co-reactant are sequentially supplied to chemisorb on the surface of a substrate, while being separated by intermittent purging steps. The half-reactions on the substrate surface will terminate automatically once the substrate surface is saturated with a (sub)monolayer of adsorbate. This allows a film to be formed with sub-nanometer thickness accuracy during each cycle. Due to the layer-by-layer deposition characteristics, the growth per cycle (GPC) is commonly used to characterize the ALD process. Typically, the GPC is of the order of one Å per cycle, depending on the individual process.^2^ Thus, the thickness of a film can be tailored by the number of cycles. For more details about this deposition process, the reader is referred to existing reviews on this topic.^2–4^

Besides controlling the film thickness at Ångström or (sub)monolayer level, another primary advantage of ALD is that it allows for conformal coating on high-aspect-ratio topologies and three-dimensionally structured substrates. This makes ALD the technology of choice over alternative deposition techniques, such as chemical vapor deposition (CVD) and physical vapor deposition (PVD), in areas where conformality is critical. In addition to film conformality, ALD can be normally conducted at lower deposition temperatures than CVD due to the different reaction mechanisms involved. Also, novel dedicated (often homoleptic) precursors have been developed with maximum thermal stability to avoid any thermally activated CVD-like reactions on the substrate, yet with increased surface reactivity. This strategy makes the ALD technique suitable for temperature-sensitive substrates, *e.g.*, polymers. A detailed comparison between ALD, CVD and PVD can be found in the reviews by Muñoz-Rojas *et al.*^5,6^

### 1.2. The main challenges of current state-of-the-art ALD

**Fig. 1:**
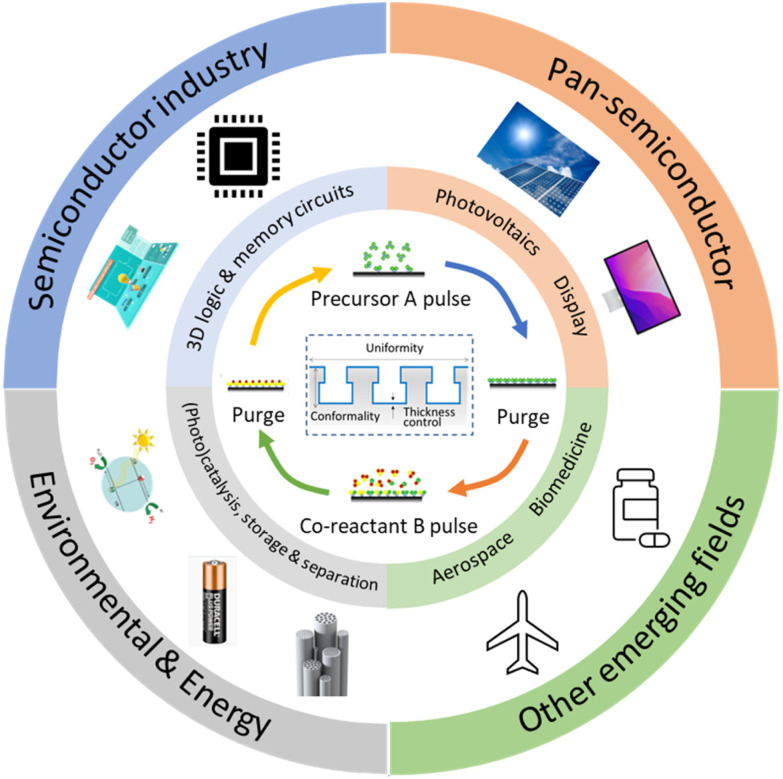
Applications of ALD in the conventional semiconductor industry and other new emerging fields. Adapted from Zhang *et al.*^9^

Given the above-mentioned merits, ALD is utilized in many other areas beyond the semiconductor industry (Fig. 1). One of the important applications of ALD technology is for energy conversion and storage purposes, for example, in the photovoltaic (PV), fuel cell and battery industry in order to achieve high device performance. These applications often require deposition of layers with nanometer-thickness on a large area.^7^ However, most ALD processes, especially temporal ALD (t-ALD), take place at low-pressure, permitting them only to be used at a large scale on high-added-value products such as semiconductors and large-area displays. Therefore, the main challenge faced by the current (temporal) ALD equipment is the high cost involved when scaling up vacuum equipment.^6^

At least as importantly, current t-ALD processes suffer from another issue: low deposition rates. Typically, the ALD rates are on the order of 100–300 nm h^−1^, which is much slower than other vapor-phase deposition methods (CVD, PVD and pulsed laser deposition (PLD)).^8^ The throughput of ALD is even lower when substrates with high aspect ratio topologies are used. The reason is that it takes more time for pulsing and purging to allow precursors to diffuse into 3D features and reaction by-products to be removed completely.^2^

### 1.3. Atmospheric-pressure ALD

**Fig. 2:**
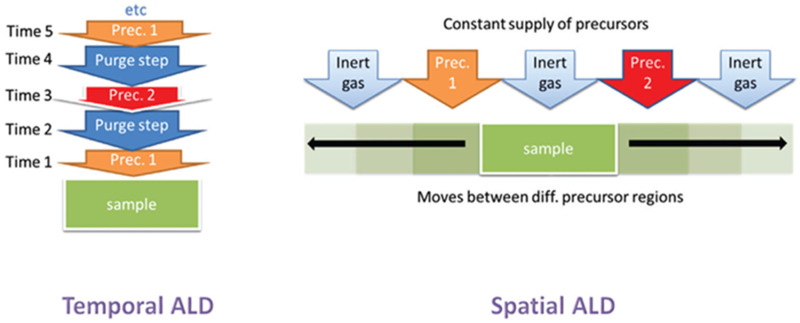
Schematic representation of t-ALD and s-ALD. From Muñoz-Rojas *et al.*^5^

In conventional t-ALD, the purging step is considered as the main limiting factor which usually accounts for up to 50% of the accumulated process time in a standard ALD cycle. In order to overcome the inherent limitation of pulsing/purging precursors into and from the reactor in a time-sequenced mode, spatial ALD (s-ALD) has emerged as a promising alternative technique that may provide high substrate throughput.^2^ Hence, minimizing or eliminating the purging time between the pulsing steps can lead to a higher effective deposition rate.^6^ Instead of the delivery of gaseous precursors to the substrate surface followed by a purging step, s-ALD operates by moving the substrate through each gas zone in succession, as shown in Fig. 2. As a consequence, the purge steps between the precursor dosage are virtually eliminated. With the spatial separation of half-reactions, instead of temporal, s-ALD can achieve time-averaged deposition rates (layer thickness per time unit) as high as 1.2 nm s^−1^, *i.e.* 4.3 μm h^−1^.^10^ Furthermore, the separation of the different reaction zones in s-ALD, typically achieved by inert gas curtains, makes it possible to operate the process at atmospheric pressure thus enabling higher throughput and lower operation cost. As a result, s-ALD has found its route towards industrial applications in areas such as PV,^11^ flexible electronics,^12^ and fuel cells.^13^ The possible advantages of atmospheric-pressure s-ALD on planar, non-porous surfaces are summarized below:

• Easier to use (no vacuum)

• Reduced cost of ownership (COO)

• Potential for easier process upscaling to large substrate areas

• Potential for higher throughput (*e.g.*, more wafers per hour)

• More flexible dopant incorporation options by co-injecting more than one precursor and more than one co-reactant at a time.

At the same time, significant research efforts have been made to reduce the ownership cost of t-ALD. In this regard, atmospheric-pressure t-ALD has been considered to make it economically viable for some emerging applications (*e.g.* porous materials).^14–16^ In the t-ALD reactor, the four sequential steps (pulse-purge-pulse-purge) compose one deposition cycle. Since layer thickness is mainly determined by the number of deposition cycles, shortening the total time needed to complete one cycle will result in increased throughput of the total ALD process. One option here, is to minimize the time needed to reach self-saturation on the substrate surface at any given reaction temperature during pulsing. To achieve this, one possible solution is to increase the flux of these precursors by using higher operation pressures, preferably atmospheric pressure. Given the short reaction time constants (typically < 0.1 s) for most half-reactions in actual film growth, the reduction of precursor and co-reactant pulse times will have a minor effect on the overall deposition rate of a t-ALD process. On the contrary, the times needed to purge out the reactive precursors and by-products by convective flow at a higher pressure can be shortened considerably in combination with a proper reactor design (*e.g.* small reactor volumes). However, when coating 3D or high-porosity substrates at high pressures, the gas diffusion in deep and narrow pores becomes slower due to Knudsen diffusion (see section 4). Thus a longer gas residence time in the high-porosity substrate is expected. In order to avoid the occurrence of an unwanted CVD reaction regime, longer purging times are needed to prevent the mixture of two reagents. Therefore, the performance of conventional ALD has been compromised with the trade-off between the long purge times to allow for full surface saturation, and minimizing the total cycle time to reach a time-efficient deposition process. By minimizing the necessary precursor gas residence times and the by-product removal times, one will reach the most optimal and cost-effective process.^17^

**Fig. 3:**
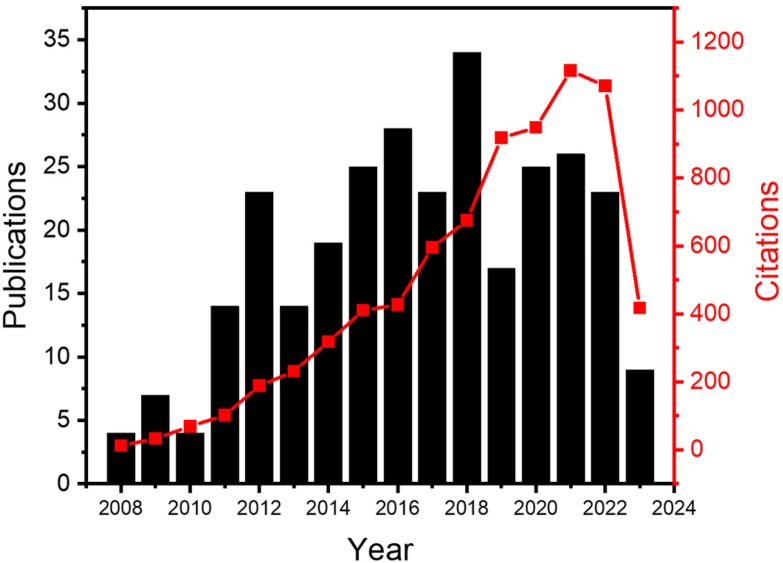
Numbers of publications and citations on the topic of atmospheric-pressure ALD from 2008 to 2023. The data were extracted from Web of Science in June 2023, with the keywords (“atomic layer deposition” OR ALD OR “molecular layer deposition” OR MLD) AND (atmospheric pressure OR “open air” OR atmospheric-pressure).

**Fig. 4:**
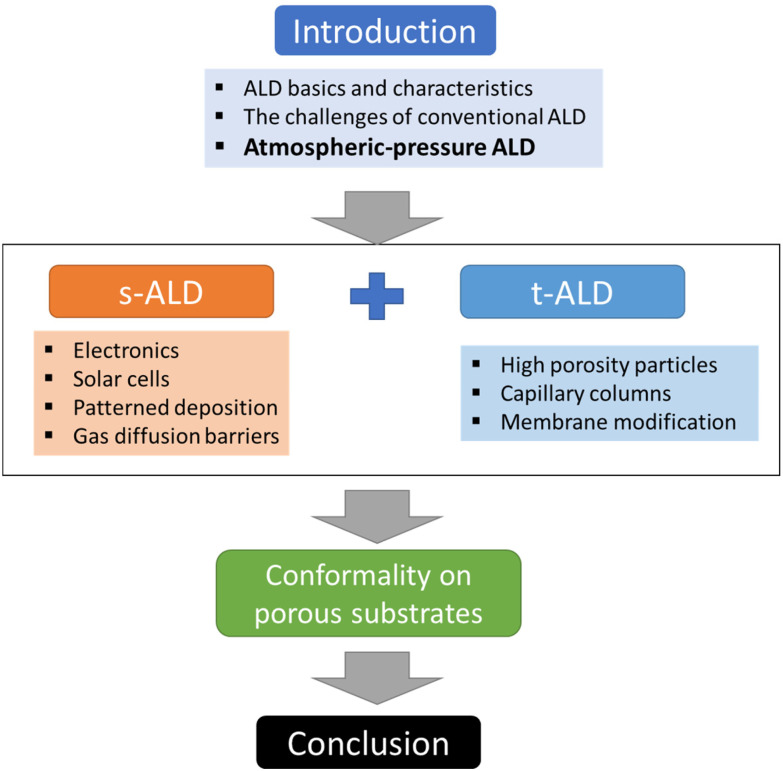
Overview of the content structure of this review, illustrating the main sections and their interrelationships (s-ALD = spatial ALD; t-ALD = temporal ALD).

In this review, we will focus on the recent developments in ALD processes operated at atmospheric pressure, or even in an open-air environment (numbers of publications and citations on this topic is given in Fig. 3). Previous reviews have been documented on atmospheric-pressure s-ALD from various points of view. For example, Poodt *et al.*^18^ provided an overview of different s-ALD reactors designed by various research institutes and companies around the world. Muñoz-Rojas and MacManus-Driscoll^11^ highlighted the potential of s-ALD in the field of low-cost PV. More recently, Muñoz-Rojas *et al.*^6^ compared the different high-throughput ALD approaches from the perspectives of processing time and targeted cost for different industries. This review aims to provide an overview of recent development and applications of both temporal and spatial atmospheric-pressure ALD and their applications in different areas (Fig. 4). The pros and cons of both ALD approaches will be compared. Finally, a future outlook on the development of atmospheric-pressure ALD will be provided.

## 2. Recent applications of atmospheric atomic layer deposition

### 2.1. A brief history of s-ALD

**Fig. 5:**
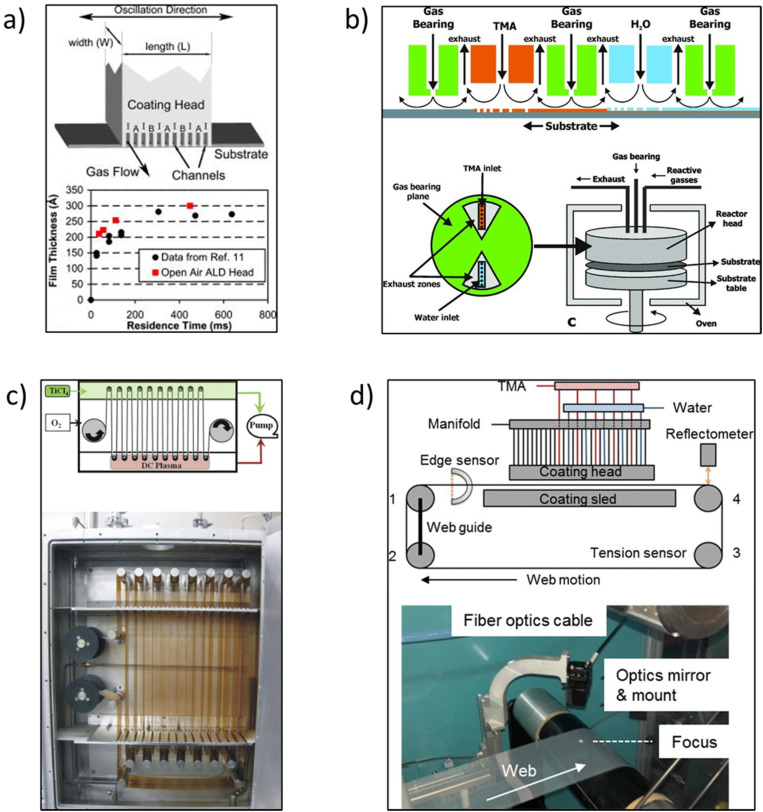
(a) Schematic of an open-air close proximity reactor. The ALD coating head shows the gas channels and gas flow. The channels are 0.7 mm wide with a spacing of 1.4 mm. The coating width *W* is approximately 50 mm. A is the oxidizing reactant, B is the metal precursor, and I is nitrogen. (b) Rotary s-ALD reactor developed by TNO. (c) R2R reactor for flexible substrates developed by Lotus Applied Technology. (d) A simple, atmospheric pressure R2R web coating s-ALD reactor. Reprinted with permission from Poodt *et al.*,^10,18^ Levy *et al.*,^22^ and Yersak *et al.*^26^

In the early times, Suntola and Antson described the original concept of s-ALD in their patent published in 1977.^19^ They designed a tool where reactants were injected at two different locations. However, the reactor still needed to be operated at low pressure with a vacuum pump. Later Suntola *et al.*^20^ proposed to use shields confining inert gas inlets to separate the reaction zones. Supported by this idea, the s-ALD concept was conceived for atmospheric pressure applications. Despite the early exploration, the development of s-ALD remained dormant for almost 25 years. In 2008, Levy *et al.*^21,22^ at Eastman Kodak reported an open-air close proximity reactor. The design of the reactor is schematically shown in Fig. 5a. In such reactor, the substrate is placed close enough (typically on the order of 50–200 μm) to an injector manifold head with alternating parallel flows of precursor gases. The precursors are fed continuously while being separated by an inert gas flow. Due to the narrow gap between the substrate and gas injection manifold, the individual reaction regions are minimized in volume and fully separated. This will strongly reduce or even eliminate precursor drag flow along the moving substrate which can cause unwanted cross-talk between the different reagents and thus counter-act monolayer thickness control. As a result, the system enables up to two orders of magnitude faster deposition rate than conventional ALD at atmospheric pressure. In addition, the close proximity approach allows the switch between a pure ALD mode and a more CVD-like mode by simply adjusting the height of the gap between the manifold and substrate. This makes the reactor design more flexible and suitable for industrial applications.

Afterwards, a new s-ALD reactor concept was developed by TNO in 2010.^10^ The group designed a rotary lab-scale reactor as schematically shown in Fig. 5b. In the reactor gas injection head, the two half-reaction zones are separated from each other by exhaust zones and gas-bearing planes. The substrate table is rotated by a servo-motor, connected by a drive shaft. The reactor head is mounted on top of the rotary table with the substrate in between, and as a whole, they are placed in a convection oven for heating. This concept was first tested to deposit ∼10 nm thin Al_2_O_3_ passivation films in solar cells. The deposition rate of Al_2_O_3_ thin films was as high as 1.2 nm s^−1^ and the layer thickness was homogeneous along the width of the deposition track. This way, the solar cells showed excellent passivation properties after the coating of the layers.^10,23^ Start-up companies have been successfully founded for the commercialization of both large-scale and pilot-scale equipment, such as Levitech, SoLayTec, and SparkNano in The Netherlands^7^ and several other companies described herebelow.

One start-up, Lotus Applied Technology developed a *roll-to-roll* (R2R) reactor for flexible substrates.^24^ In this configuration, the two precursor zones are separated by an inert gas which also works as a purging zone (Fig. 5c). One of the main advantages of this design is that it can serve for large-area flexible device applications. The first demonstration was the deposition of moisture barrier films of Al_2_O_3_ and TiO_2_ on 100 mm wide polyethyene terephthalate (PET) foils that moved with a speed of over 1 m s^−1^. Another type of R2R reactor was developed by Maydannik *et al.*^25^ In their approach, a rotating drum was used as a support for the flexible web substrate, placed in a heated cylindrical chamber. The web was exposed to various inlet gas and purge zones with one rotation being equivalent to one ALD cycle. It was demonstrated that flexible substrates with a width of 300 × 100 mm^2^ could be coated uniformly. However, these R2R reactor concepts are operated at a pressure of about 2 mbar, similar to that in conventional ALD. This low pressure was used to guarantee the individual reaction zones.

To take R2R ALD one step further in scaling up at reduced capital cost, Yersak *et al.*^26^ developed a simple, atmospheric pressure R2R web coating system in 2014. The configuration of this web coating system is shown in Fig. 5d. A modular ALD gas source head was designed with the first 10 nozzles filled with N_2_, followed by sets of precursors and N_2_ bands. The polymer used in the study was polyethylene naphthalate (PEN) with foil length and width dimensions of 4 m × 100 mm. The PEN web was fastened on four rollers with Kapton tape and circulated in a loop using a belt-driven motor system. Sustained by computational fluid dynamics (CFD) modelling, the appropriate deposition parameters could be first identified. In addition, a spectral reflectometer was mounted onto the injection module to measure the layer thickness and uniformity *in situ*. Al_2_O_3_ was then grown at 100 °C at a rate of 0.11–0.13 nm per cycle, with ALD cycle times of 76 ms and a web speed of 1 m s^−1^. Later, Ali *et al.*^27,28^ also designed a similar R2R system working at atmospheric pressure. A movable web of PET was used as the substrate onto which Al_2_O_3_ was deposited at 50 °C at a web speed of 7 mm s^−1^. The deposited films had a low roughness and good chemical, electrical and optical properties.

The use of water as a co-reactant is common in most ALD processes as it is inexpensive and easily available. However, its vapor pressure and high sticking coefficient can also cause issues such as low reaction kinetics and, in particular, the need for longer purging times. In addition, polymer substrates are very sensitive to temperature. Deposition at low temperature (*e.g.*, room temperature) is preferred in this case. To this end, researchers from TNO started incorporating a dedicated low-damage DBD (dielectric barrier discharge) plasma source into the s-ALD reactor at atmospheric pressure in 2011. This way, Al_2_O_3_ films could be deposited at 150 °C using trimethyl aluminium (TMA) in combination with such a DBD plasma-enhanced O_2_ source.^29^ By switching on and off the plasma at specific locations, 2D deposition patterns were created. Later, the University of Wuppertal in cooperation with the company SENTECH Instruments also demonstrated a home-built atmospheric-pressure s-ALD equipped with a DBD source.^30^ The authors deposited TiO_*x*_ at room temperature with a growth rate comparable to the one reported for low-pressure plasma-enhanced ALD (PE-ALD) or sol–gel processing. The minor contribution of CVD to the growth rate was supposed to be caused by the residual water present in the system.

Integrated circuits (IC) are the backbone of micro- and nanoelectronic devices. One driving force to favor the high-throughput s-ALD concept in commercial IC mass production would certainly be the lower cost.^7^ Jusung Engineering^31^ was one of the very first companies to introduce low-pressure s-ALD to the semiconductor industry. Using their advanced “Cyclone” s-ALD reactor they realized 3D multiple (MIMIM) capacitor layer stacks of TiN/Al_2_O_3_/TiN/Al_2_O_3_/TiN deposited on an (arsenic) n^++^-doped Si-substrate with macropore arrays etched with ∼1.5 μm pore diameter and 30 μm pore depth.^32^ TiN layers were deposited at 400 °C at 500 mTorr from TiCl_4_ and NH_3_ vapor dosing using sub-second pulse-purge exposures. To avoid oxidation of the TiN electrode layers and thus maintaining the conductivity of the electrodes (<200 μΩ cm), the Al_2_O_3_ layers were grown in a separate chamber without breaking the vacuum using PE-ALD in O_3_ from TMA at 380 °C and 1.2 Torr and 1.2 Torr, using TMA/purge/O_3_/purge exposures of 0.5/2/3/2 seconds. Later, the low-pressure s-ALD market was dominated by Tokyo Electron (TEL) with their NT-333™ semi-batch reactor.^33^ In subsequent years, similar reactor designs were realized by several other companies such as Applied Materials,^34–36^ Lotus Applied Technology,^37^ Beneq Oy,^38^ Wonik IPS^39^ and NCD.^40^Table 1 gives a short overview of s-ALD reactors offered commercially.

**Table 1:** Overview of a selection of commercially available low-pressure s-ALD reactors. For an extensive recent overview see ref. 41

Company	s-ALD reactor name
Applied Materials	Olympia™
Beneq Oy	Beneq C2R
Jusung Engineering	Cyclone
Lotus Applied Technology	Vortex ALD™
NCD	Lucida™ S
Tokyo Electron (TEL)	NT-333™
Wonik IPS	HyEta™

### 2.2. Applications of s-ALD

**Fig. 6:**
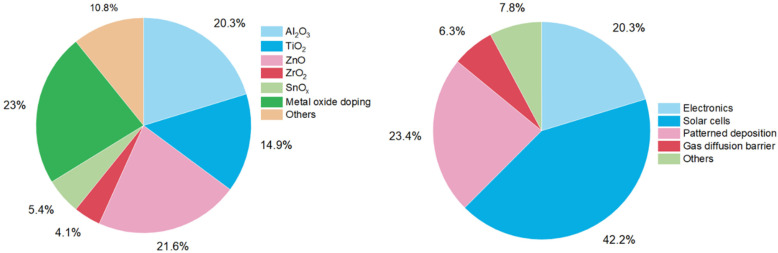
Pie chart distributions of atmospheric-pressure ALD studies conducted in 2008–2023 on materials (left) and applications (right).

Besides electronics, more application fields for atmospheric-pressure s-ALD are emerging. One application both in academia and industry, with low-cost potential, is the deposition of passivation layers on large-area substrates in the solar (PV) industry. Applications in display technology are in gas diffusion barriers for moisture-sensitive devices, *e.g.*, organic light-emitting diodes (OLEDs),^42^ and in next-generation high-mobility amorphous semiconductor oxides such as In_*x*_Ga_*y*_Zn_*z*_O (IGZO),^43,44^ enabling ease of scalability and compatibility with R2R operation mode. An overview of more emerging applications of atmospheric ALD processes is given in Table 2. The pie chart distributions of publications dedicated to each material and application are shown in Fig. 6.

**Table 2:** Overview of atmospheric-pressure ALD processes in present applications

Materials	Precursor	Co-reactant	Substrate	Deposition temperature	Applications	ALD type	Year	Ref.
ZnO	DEZ	NH_3_/H_2_O/O_2_	Glass coated with Cr and Al_2_O_3_ film	250 °C	TFTs	s-ALD	2008	22
Al_2_O_3_	TMA	H_2_O	Silicon	200 °C	TFTs	s-ALD	2009	45
ZnO	DEZ	NH_3_/H_2_O/O_2_	Glass coated with Cr and Al_2_O_3_ film	—	TFTs	s-ALD	2009	45
ZnO	DEZ	H_2_O	Silicon/glass coated with Cr	100–250 °C	TFTs	s-ALD	2012	46
IGZO	TMIn/TEGa/DEZ	H_2_O	Silicon	200 °C	TFTs	s-ALD	2015	43
IZO	TMIn/DEZ	N_2_/O_2_	Silicon	160 °C	TFTs	PE-s-ALD	2018	47
IGZO	TMIn/TEGa/DEZ	N_2_/O_2_	Glass	160 °C	TFTs	PE-s-ALD	2019	48
SnO_2_	Sn(acac)_2_	H_2_O	Glass	270 °C	TFTs	s-ALD	2022	49
Al_2_O_3_	TMA	O_3_	Silicon	100–200 °C	TFTs	PE-s-ALD	2022	50
SnO	Sn(TAA)_2_	H_2_O	Silicon	100–210 °C	TFTs	s-ALD	2022	51
ZnO	DEZ	H_2_O	Glass	50–200 °C	Optoelectronic	s-ALD	2022	52
TiO_2_	TiCl_4_	H_2_O	Glass	150 °C	Optoelectronic	s-ALD	2022	52
Al_2_O_3_	TMA	H_2_O	Glass	100–200 °C	Optoelectronic	s-ALD	2022	52
N-doped ZnO	DEZ	NH_3_/H_2_O	Silicon	100–300 °C	Solar cell	s-ALD	2008	53
Al_2_O_3_	TMA	H_2_O	Silicon	200 °C	Solar cell	s-ALD	2010	10
Al_2_O_3_	TMA	H_2_O	Silicon	200 °C	Solar cell	s-ALD	2011	54
TiO_*x*_	TIP	Ar/O_2_	Silicon/glass	RT	Solar cell	PE-s-ALD	2013	30
ZnO	DEZ	NH_3_/H_2_O	ITO/glass	150 °C	Solar cell	s-ALD	2013	55
TiO_2_	TiCl_4_	H_2_O	ITO/glass	100/350 °C	Solar cell	s-ALD	2013	56
ZnOS	DEZ	H_2_O/H_2_S	Silicon	120	Solar cell	s-ALD	2015	57
AZO	DEZ/TMA	H_2_O	Silicon	200 °C	Solar cell	s-ALD	2015	57
Zn_1−*x*_Mg_*x*_O	DEZ/Mg(CpEt)_2_	H_2_O	Cu_2_O	—	Solar cell	s-ALD	2015	58
ZnOS	DEZ	H_2_O/H_2_S	Silicon	120 °C	Solar cell	s-ALD	2016	59
ZnO	DEZ	H_2_O	ITO/glass	—	Solar cell	s-ALD	2016	60
ZnO	DEZ	H_2_O	Cu_2_O	150 °C	Solar cell	s-ALD	2016	61
ZnMgO	DEZ/Mg(CpEt)_2_	H_2_O	Cu_2_O	150 °C	Solar cell	s-ALD	2016	61
ZnO	DEZ	H_2_O	Glass	250 °C	Solar cell	s-ALD	2017	62
AZO	DEZ/TMA	H_2_O	Glass	250 °C	Solar cell	s-ALD	2017	62
Al_2_O_3_	TMA	H_2_O	ZnO nanorod arrays	100–350 °C	Solar cell	s-ALD	2017	63
ZnO	DEZ	H_2_O	ZnO nanorod arrays	100–350 °C	Solar cell	s-ALD	2017	63
Cu_2_O	Cupraselect	H_2_O	ZnO nanorod arrays	100–350 °C	Solar cell	s-ALD	2017	63
N-doped ZnO	DEZ	30% NH_3_ in H_2_O	ZnO nanorod arrays	100–350 °C	Solar cell	s-ALD	2017	63
Zn_1−*x*_Mg_*x*_O	DEZ/Mg(CpEt)_2_	H_2_O	ZnO nanorod arrays	100–350 °C	Solar cell	s-ALD	2017	63
ZnO	DEZ	H_2_O	Silicon/glass	95 °C	Solar cell	s-ALD	2018	64
ZnOS	DEZ	H_2_O/H_2_S	Silicon/polyamide foil	100–200 °C	Solar cell	s-ALD	2018	65
SnO_*x*_	TDMASn	H_2_O	Glass	80–150 °C	Solar cell	s-ALD	2018	66
NiO_*x*_	Ni(MeCp)2	O_2_	Glass	350 °C	Solar cell	s-ALD	2018	67
Cu_2_O	CuhfacTMVS	H_2_O	Silicon	200/220 °C	Solar cell	s-ALD	2021	68
TiO_2_	TTIP	H_2_O	Glass	100–300 °C	Solar cell	s-ALD	2021	69
TiO_2_	TiCl_4_	H_2_O	Glass	100–300 °C	Solar cell	s-ALD	2021	69
Al_2_O_3_	TMA	He/O_2_	Silicon	150 °C	Patterned deposition	PE-s-ALD	2011	29
ZnO	DEZ	H_2_O	Silicon/glass	100–300 °C	Patterned deposition	AS-s-ALD	2014	70
Al_2_O_3_	DMAI	H_2_O	Silicon/glass	100–300 °C	Patterned deposition	AS-s-ALD	2014	70
AZO	DEZ/DMAI	H_2_O	Silicon/glass	100–300 °C	Patterned deposition	AS-s-ALD	2014	70
ZnO	DEZ	H_2_O	—	200 °C	Patterned deposition	s-ALD	2019	71
TiO_2_	TTIP	N_2_/O_2_ plasma	Silicon/glass	65 °C	Patterned deposition	PE-s-ALD	2019	72
ZnO	DEZ	H_2_O	3D printed substrates	—	Patterned deposition	s-ALD	2020	73
TiO_2_	—	—	3D printed substrates	—	Patterned deposition	s-ALD	2021	74
TiO_2_	TTIP	H_2_O	—	—	Patterned deposition	s-ALD	2021	75
ZnO	DEZ	H_2_O	—	—	Patterned deposition	s-ALD	2021	75
TiO_2_	TTIP	H_2_O	Silicon/aluminum/glass	150 °C	Patterned deposition	s-ALD	2021	76
ZrO_2_	ZTB	H_2_O	Silicon/aluminum/glass	150 °C	Patterned deposition	s-ALD	2021	76
TiO_2_	TTIP	H_2_O	Silicon	120–260 °C	Patterned deposition	s-ALD	2022	77
Pt	(MeCp)PtMe_3_	O_3_	Silicon	200–250 °C	Patterned deposition	s-ALD	2022	77
SiO_2_	BDEAS	Ar/O_2_	Silicon *vs.* ZnO/silicon	100 °C	Patterned deposition	AS-s-ALD	2023	78
Al_2_O_3_	TMA	H_2_O	PET	50 °C	Gas diffusion barrier	s-ALD	2014	28
Al_2_O_3_	TMA	Ar/O_2_	Silicon/PET	80 °C	Gas diffusion barrier	PE-s-ALD	2016	79
Al_2_O_3_	TMA	Ar/O_2_	Silicon/glass	75–150 °C	Gas diffusion barrier	PE-s-ALD	2017	80
SnO_*x*_	TDMASn	O_2_/O_3_/H_2_O	Silicon/glass	80–165 °C	Gas diffusion barrier	PE-s-ALD	2018	81
ZnO	DEZ	H_2_O	Glass	200 °C	Sensors	s-ALD	2018	82
AZO	DEZ/TMA	H_2_O	Glass	200 °C	Sensors	s-ALD	2018	82
Al_2_O_3_	TMA	H_2_O	Window glass	50 °C	Wind shields	t-ALD	2015	83
TiO_2_	TDMAT	O_3_	PDMS	100 °C	Chemical stability	t-ALD	2022	84
ZrO_2_	TEMAZ	N_2_/O_2_	Silicon/glass	150–250 °C	MIMC	PE-s-ALD	2017	85
Al_*x*_Zn_1−*x*_O	DEZ/TMA	H_2_O	Glass	200 °C	—	s-ALD	2013	86
IZO	TMIn/DEZ	H_2_O	Glass	200 °C	—	s-ALD	2014	87
Ag	Ag(fod)(Pet_3_)	N_2_/H_2_	Silicon	100–120 °C	—	PE-s-ALD	2015	88
Al_2_O_3_	TMA	N_2_/O_2_	Silicon	20–100 °C	—	PE-s-ALD	2016	89
ZrO_2_	TEMAZ	N_2_/O_2_	Silicon	20–100 °C	—	PE-s-ALD	2016	89
Al_2_O_3_	TMA	H_2_O	Quantum dot films	27 °C	—	t-ALD	2016	90
Ag	(NHC)Ag(hmds)	Ar/H_2_	—	100 °C	—	PE-s-ALD	2018	91
Al_2_O_3_	Al(CH_3_)_3_	Ar/O_2_	Silicon	80 °C	—	PE-s-ALD	2019	92
ZnO	DEZ	H_2_O	Glass	200 °C	—	s-ALD	2021	93
Alucone	DMAI	EG	Silicon	200 °C	—	MLD	2022	94

#### 2.2.1. Electronics

One area in which s-ALD received the most intensive attention is thin-film electronics. In particular, thin-film transistors (TFTs) are recognized as a potential application field for next-generation large-area electronics (*e.g.*, for TFT display applications) as the films can be deposited on diverse substrates, over large areas, and at reasonably low process temperatures.^95^ To meet the needs of the fabrication of TFTs, s-ALD proved to be the preferred option in terms of film quality, uniformity and thickness control compared to other deposition methods such as sputtering,^96,97^ PLD^98^ and solution processing.^99^ In addition, the operation at atmospheric pressure makes s-ALD more economically competitive than other, vacuum-based, systems.

Compared to other mixed metal oxides, ZnO-based oxides, especially amorphous semiconducting oxides such as IGZO, are the most widely studied for next-generation TFTs due to features including high mobility, excellent electric and chemical stability and the possibility for doping.^45,100^ The first study of ZnO-based TFTs by atmospheric-pressure s-ALD was authored by Levy *et al.*^22,45^ They deposited ZnO on top of the Al_2_O_3_ dielectric by a close-proximity s-ALD reactor. The ZnO-based TFTs thus prepared showed good performance with electron mobility approaching 20 cm^2^ V^−1^ s^−1^ and excellent stability. Later, the same group investigated the effect of cycle times on growth and transistor characteristics of ZnO deposited by atmospheric-pressure s-ALD. Once more, purge times are critical in optimizing the mobility of the TFT device. Electron mobility data as high as 22 cm^2^ V^−1^ s^−1^ have been realized for the ZnO-based TFTs grown at 200 °C with purge times as short as 25 ms without indication of a CVD component being present.^46^

In order to optimize and tailor the electronic properties of ZnO-based devices, doping is considered as an easy and efficient way achieved by atmospheric-pressure s-ALD.^100^ In s-ALD, doping can be easily realized by co-injecting the vaporized metal precursors into the deposition region. By adjusting the ratio of precursors, the properties of the resulted multicomponent oxides can be finely tuned. Commonly used dopant atoms for producing ZnO-based mixed oxide electronics are Al,^86^ In,^87^ N,^95^ and Mg.^101^ For example, Hoye *et al.*^101^ developed fluorene-free perovskite organometal halide light-emitting diodes (LEDs) by replacing the F8 electron injector with Mg-incorporated ZnO. The obtained LEDs had a higher luminance and lower turn-on voltages due to the reduced electron injection barrier of ZnO. By doping N in ZnO, Nelson *et al.*^46^ increased the sheet resistance of ALD-grown ZnO layers by more than an order of magnitude. To improve the mobility of TFTs, Illiberi *et al.*^47^ prepared indium zinc oxide (InZnO) using plasma-enhanced atmospheric-pressure s-ALD with trimethyl indium (TMIn), diethyl zinc (DEZ) and deionized water as precursors. The In/(In + Zn) ratio of the film was accurately tuned by varying their flows. The TFTs obtained at an In/Zn ratio of 2 : 1 showed a high mobility of over 30 cm^2^ V^−1^ s^−1^ and excellent stability.

The same authors also investigated the doping of both In and Ga into ZnO to prepare an amorphous oxide semiconductor (AOS). Amorphous IGZO is recognized as a promising material for the active channel in TFTs. The metal composition of IGZO was controlled by varying the flow of precursors. It was found that Ga-ions could hinder the formation of oxygen vacancies, suppressing the generation of free carriers and thus a decrease in field effect mobility with increased Ga-content was observed.^43^ Later, Katsouras *et al.*^48^ demonstrated the uniform deposition of IGZO on large substrates *via* the integration of an s-ALD-deposited Al_2_O_3_ buffer layer into the TFT stack. The final products showed low off-currents and field effect mobility of 9 cm^2^ V^−1^ s^−1^. More recently, Yoo *et al.*^50^ improved the performance of IGZO TFTs deposited on a polyimide substrate with an s-ALD-derived Al_2_O_3_ layer as the gate insulator. The field effect mobility of the IGZO TFT is as high as 52.5 cm^2^ V^−1^ s^−1^, while maintaining excellent bias reliability and mechanical bending stability.

Other mixed metal oxides have also been deposited with atmospheric-pressure s-ALD and explored as potential AOS materials for TFTs. For example, Mameli *et al.*^51^ reported the fabrication of p-type SnO-based TFTs with tin(ii)-bis(*tert*-amyloxide), Sn(TAA)_2_, and H_2_O as the co-reactant by atmospheric-pressure s-ALD. Compared to conventional t-ALD, the deposition rates of SnO were up to 19.5 times higher when using atmospheric-pressure s-ALD. However, the linear mobility of the TFTs was only 0.4 cm^2^ V^−1^ s^−1^, which is lower than for the one deposited by t-ALD.^102^ Nguyen *et al.*^49^ demonstrated the deposition of SnO_2_ thin films by atmospheric-pressure s-ALD with tin(ii) acetylacetonate (Sn(acac)_2_) and water as precursors. Due to the n-type nature of as-deposited SnO_2_, a high electron mobility of the films up to 11.2 cm^2^ V^−1^ s^−1^ was achieved.

#### 2.2.2. Solar cells

Solar energy is regarded as one of the most promising ways to tackle today's global energy issues and environmental challenges by replacing carbon-intensive sources.^103^ Today, the PV market is largely dominated (∼90%) by multicrystalline silicon solar cells, for several reasons.^104,105^ First, silicon is the world's second most abundant element with a suitable bandgap, making it technically and economically feasible not only as a mainstay material in semiconductor electronics but also in PV, the latter with energy conversion efficiency values up to 25% recorded in 1999^106^ with an intrinsic limit of 29%.^105^

To maximize their efficiency, solar cells are usually coated with an Al_2_O_3_ passivation layer with a proper thickness of typically ∼10 nm. Also, to realize mass production of solar cells for practical applications, high-throughput manufacture in a continuous process is a strict prerequisite.^107^ Here, the characteristics of low-cost, high-throughput and ease of scalability make atmospheric-pressure s-ALD an ideal candidate to fulfil these requirements in the PV industry.

Until recently, several thin-film materials have been tested and industrially employed for surface passivation of silicon solar cells such as silicon dioxide,^108^ silicon nitride^109^ and amorphous silicon.^110^ Compared to those materials, Al_2_O_3_ was demonstrated to be superior in increasing the efficiency of silicon solar cells drastically, as shown by the t-ALD work of Hoex and co-authors.^111^ This excellent surface passivation was attributed to the high negative fixed charge formed at the silicon/Al_2_O_3_ interface. Later, Poodt *et al.*^54^ demonstrated a high-quality and high-rate deposition of Al_2_O_3_ passivation films on silicon solar cells. Using atmospheric-pressure s-ALD they achieved extremely high growth rates up to 1.2 nm s^−1^ of the Al_2_O_3_ layers with excellent surface passivation. This concept has been commercialized by SoLayTec into a modular high-volume manufacturing tool, designed with 6 to 8 chambers yielding throughput numbers of more than 4000 wafers per h to meet industrial cost-efficiency requirement.^54^ Besides Al_2_O_3_ as a passivation cap layer, other functional materials have also been explored by atmospheric-pressure s-ALD to boost the efficiency of silicon solar cells. For example, Nguyen *et al.*^68^ studied the integration of Cu_2_O films as a hole-transporting layer in silicon heterojunction solar cells. The low-temperature deposited Cu_2_O films realized a power conversion efficiency of 13.7%, higher than other relevant studies. Here, the purity and stoichiometry of Cu_2_O is considered as key in maximizing the efficiency of solar cells since the presence of surface CuO was found to be detrimental.^112^

Beyond crystalline silicon, atmospheric-pressure s-ALD has also attracted the attention for the production of next-generation PV cells including CuInGaS (CIGS), organic and perovskite solar cells. The low production cost and high conversion efficiency (>21%) of CIGS solar cells make them gain increasing attention as the 2^nd^ generation of PV. Typically, a buffer layer like CdS is used in CIGS solar cells to improve the overall efficiency. However, Cd is toxic and this drives researchers to explore other alternative materials. Zn(O,S), with a wider band gap than CdS, is considered an ideal replacing material. Interesting work on mixed oxysulfide (Zn(O,S)) functional layers with atmospheric-pressure s-ALD was published by Illiberi *et al.*^57^ They deposited Zn(O,S) buffer layers by exposing the substrate simultaneously to both H_2_O and H_2_S precursors, which were pre-mixed and co-injected in the same deposition zone. The effect of the S/(S + O) ratio on the optoelectronic and morphological properties of the Zn(O,S) layers was reported. A cell efficiency up to 15.9% was achieved at an optimum S/(S + O) ratio of about 0.4.^59^ This success prompted the researchers to transfer the Zn(O,S) deposition process to an industrial R2R s-ALD setup. An array of mini-modules with a surface area of 270 cm^2^ on a flexible substrate was demonstrated with a cell efficiency of 9.2%.^65^ The main advantage of organic solar cells lies in the flexibility of polymers, making them mass-manufacturable on various flexible substrate materials.^113^

Theirich *et al.*^30^ reported on the deposition of TiO_*x*_ by plasma-enhanced atmospheric-pressure s-ALD at room temperature as the interlayer in inverted organic solar cells which performed similarly to those containing TiO_*x*_ films prepared by conventional ALD or sol–gel processing.

Recently, research interest in solar cells has shifted towards perovskite-type cells due to their potential of reaching high energy conversion efficiency and low fabrication costs.^64^ Atmospheric-pressure s-ALD combined with R2R compatibility, would make up for a unique technology option.^7^ Najafi *et al.*^64^ employed a ZnO buffer layer to enhance the electron extraction in a perovskite solar cell structure. Improved efficiency and stability of the device were observed. Hoffmann *et al.*^66^ demonstrated ALD-grown SnO_*x*_ as impermeable electron extraction layers for perovskite solar cells. The optical transmittance and electrical conductivity of the layer were similar to those reported for conventional ALD-grown layers.

#### 2.2.3. Patterned deposition

Lithographic patterning is one of the most widely used techniques in the semiconductor industry. However, it involves a repetitive sequence of process steps, making it costly and complex for device fabrication. In recent years, area-selective s-ALD (AS-s-ALD) has emerged as a promising solution for surface patterning deposition in device manufacturing. This approach offers significant potential for precise and controlled deposition on selected areas, opening up new possibilities for advanced fabrication processes.^114^ In this technique, surface patterning is achieved by pretreating a specific part of the substrate surface through the application of an inhibitor material. This coating renders the treated area inert towards a particular ALD process, enabling precise control over the pattern formation.^70^ Alternatively, thin film patterns can be directly deposited on a substrate using plasma-enhanced ALD, where film growth is limited to localized areas exposed to the plasma.^29,72^ More recently, researchers have demonstrated the possibility to direct surface patterning *via* a simple versatile miniaturized open-air s-ALD head, which is developed by additive manufacturing.^71,73,76^ With such a technique, true direct printing of complex patterns with a lateral resolution of sub-millimeters is successfully achieved in a miniaturized nozzle. One example is the ATLANT3D™ technology developed by ATLANT3D.^74,75,77^

#### 2.2.4. Gas diffusion barriers

Encapsulation of displays, *e.g.*, OLED, with gas diffusion barriers is mandatory to protect the device from harmful ambient moisture and oxygen. To meet this objective, a low pinhole density and conformal thin film coated on the substrate surface is strictly desired. In addition, to realize the large-area, low-cost mass production of thin films in practice, s-ALD, especially the R2R atmospheric ALD, is considered as one of the best options. Ali *et al.*^115^ studied the deposition of Al_2_O_3_ films as gas diffusion barriers on a PET substrate with R2R s-ALD at near-atmospheric pressure. A water vapor transmission rate (WVTR) of ∼10^−3^ g m^−2^ day^−1^ at 37.8 °C/100% relative humidity has been reported for layers with nanometer thickness (15–40 nm). Hoffmann *et al.*^79^ also demonstrated the growth of Al_2_O_3_ films on indium tin oxide coated PET with atmospheric PE-ALD. The WVTR was as low as 3.1 × 10^−5^ g m^−2^ day^−1^ tested in a climate chamber (50 °C and 60% relative humidity). Later, Hoffmann and co-workers^81^ prepared transparent conductive gas diffusion barriers based on thin films of SnO_*x*_ with high electric conductivity (10^−4^ (Ω cm)^−1^), and a low WVTR down to 7 × 10^−4^ g m^−2^ day^−1^ at 60 °C and 60% relative humidity. These studies have shown that (plasma-enhanced) s-ALD is an excellent candidate for the continuous production of gas diffusion barriers for moisture and oxygen sensitive devices.

**Fig. 7:**
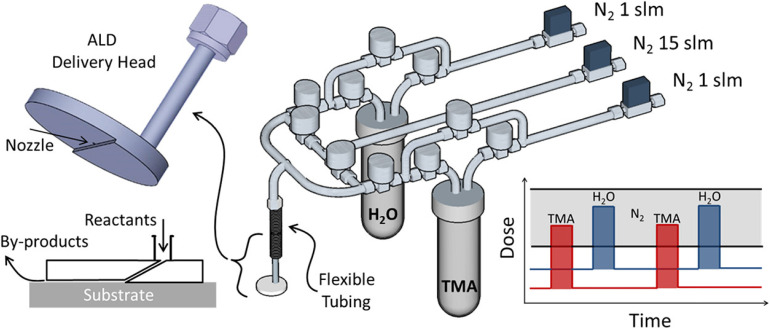
Atmospheric t-ALD with a disk-shaped delivery head in an open-air environment. Reprinted with permission from Mousa *et al.*^116^ Copyright 2015, American Chemical Society.

Besides s-ALD, atmospheric t-ALD has also been demonstrated for several applications with planar substrates. One intriguing example is the deposition of Al_2_O_3_ films on the windshield of an automobile with a disk-shaped ALD delivery head in an open-air environment (Fig. 7).^116^ Another example is the coating of TiO_2_ films onto polydimethylsiloxane (PDMS) to enhance the organic solvent resistance of the materials.^84^

## 3. New emerging applications of atmospheric-pressure atomic layer deposition

### 3.1. Considerations for atmospheric-pressure temporal ALD

One of the first reports on the deposition of thin films using t-ALD at atmospheric pressure dates back to 1988. Hunter and Kitai^117^ utilized dimethylzinc (DMZ) and hydrogen sulfide (H_2_S) as the two precursors, respectively, to deposit ZnS on a silicon substrate, and demonstrated that t-ALD can be operated without a vacuum pump to produce stoichiometric films of high purity and crystal quality.

In s-ALD, the half-reaction zones are separated spatially, making the purging steps between the precursor dosages virtually obsolete.^18^ However, this cannot be readily achieved in t-ALD.

**Fig. 8:**
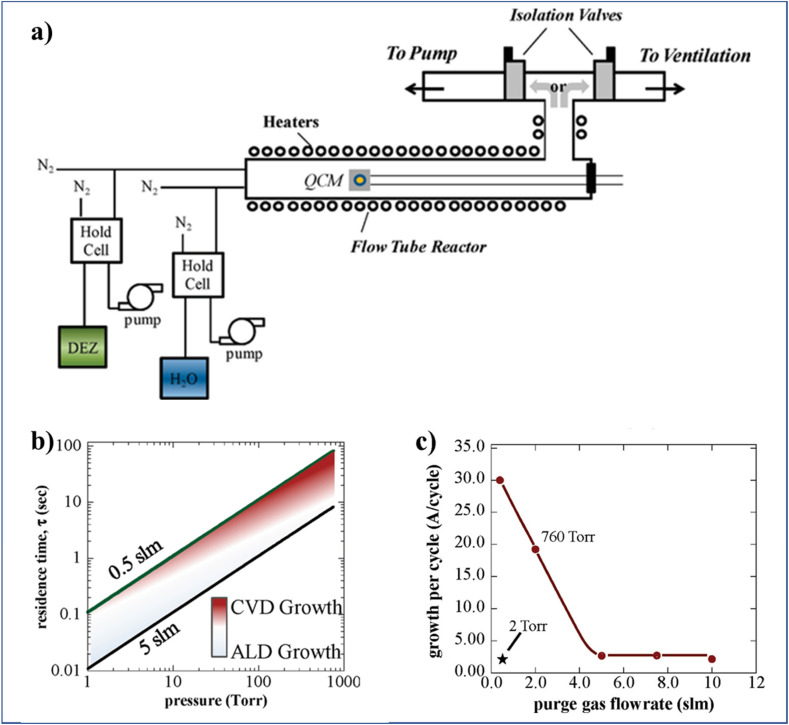
(a) Schematic of a flow tube ALD reactor (60 cm length and 3.8 cm inner diameter) design which allows for the deposition at pressures varying from vacuum to atmospheric, (b) residence time *vs.* operating pressure of the flow tube, and (c) GPC of ZnO at 760 and 2 Torr, using 30 s purge times *vs.* purge gas flow rate. Reprinted with permission from Jur *et al.*^14^ Copyright 2011, American Chemical Society.

To increase the accumulated time-averaged deposition rates in t-ALD at atmospheric pressure, one trivial strategy is to drastically reduce the reactor volume, from liters (usual in t-ALD) to only milliliters in s-ALD, by narrowing the gap between the gas injection head to the substrate down to ∼100 to 200 μm. An additional measure to further prevent the mixing of precursors here is to increase the gas flow rate (for a minimized reactor volume). To this end, Jur and Parsons^14^ designed a unique ALD system in a flow tube geometry (with 60 cm length and 3.8 cm inner diameter) which offered the ability to adjust the process pressures independently and fixed at values between 2 and 760 Torr (Fig. 8a). To ensure sufficient exposure to the precursors during each self-limiting half-reaction under the high pressure and flow rate conditions, two separate inert gas streams were utilized. One stream pushes the precursors through a hold cell into the reactor at a fixed rate of 0.5 slm, while the other flows directly into the reactor to control the gas residence time. Based on simple gas kinetic models, a plot of gas residence time as a function of reactor pressure was obtained for two different values of gas flow rate (Fig. 8b). As expected, a transition from ALD (light shading) to CVD (dark area) region was observed. The analysis indicated that it is still possible to maintain good quality ALD regime at a pressure of 760 Torr by increasing the gas flow rate up to 5 slm. In addition, the authors illustrated experimentally the effect of the gas flow rate on GPC for the deposition of ZnO at 760 Torr (Fig. 8c). At atmospheric pressure and high gas flow (5 slm), the GPC of ZnO was the same as typical for the low-pressure ALD case. However, the GPC of Al_2_O_3_ was slightly larger than that at low pressure.

### 3.2. Emerging applications of atmospheric-pressure ALD on high-porosity and/or 3D materials

In section 2.2, we have shortly discussed the recent applications of atmospheric ALD on substrates which typically have planar and non-porous structures. Atmospheric-pressure ALD also allows for the deposition on three-dimensional (3D) substrates with high uniformity and conformality. In this section, we will focus on new emerging applications on highly porous and/or 3D materials. One of the foremost applications is in high-porosity particle coating. These ALD-modified particles have many important applications in catalysis, pharmaceuticals and energy conversion.^118^ In addition, atmospheric-pressure ALD has been demonstrated as feasible for functionalizing capillary columns for gas chromatography, and membrane modification and functionalization for water treatment and gas purification.

#### 3.2.1. High-porosity particle coating

The unique capability of ALD to provide conformal coatings with sub-nanometer control makes it an ideal technique for powder modification over conventional wet-chemistry approaches.^134^ Effective coating of individual nanoparticles is, however, still facing several challenges such as their high specific surface area and agglomeration, and diffusion limitation.^135^ In particular, agglomeration, due to inter-particle forces (*e.g.*, van der Waals forces), is considered as the main limiting factor affecting the overall coating quality during ALD processes. For example, it takes a very long time for the precursors to diffuse into the bulk of agglomerates, especially in a static particle bed reactor. Also, the heat and mass transport rates between the gaseous precursors and porous, solid particles are low, hampering uniform coatings on the particle surface. To improve the efficiency of heat and mass transport, fluidized-bed ALD reactors have been developed to enhance the gas–solid interactions *via* a better dispersion of cohesive particles. The earliest work on particle coatings with fluidized-bed ALD reactors was carried out by Hakim *et al.*^136^ in 2005. In their study, Al_2_O_3_ nanolayers were conformally coated on the surface of ∼26 nm-sized zirconia nanoparticles while the particle size distribution and surface area were not affected by the coating process. Afterwards, successful particle coating utilizing fluidized-bed ALD reactors has been reported in literature for many practical cases. This makes it the most prevailing reactor type to achieve mass production for a variety of materials. In a fluidized bed reactor design, an inert gas flow (*e.g.*, nitrogen) is typically used to suspend the particles. The gas flow should be carefully selected. At too high flows, the particles can be blown out from the top of the column. Whereas, a too small gas flow cannot ensure a uniform distribution of particles along the entire column height.^137^

**Fig. 9:**
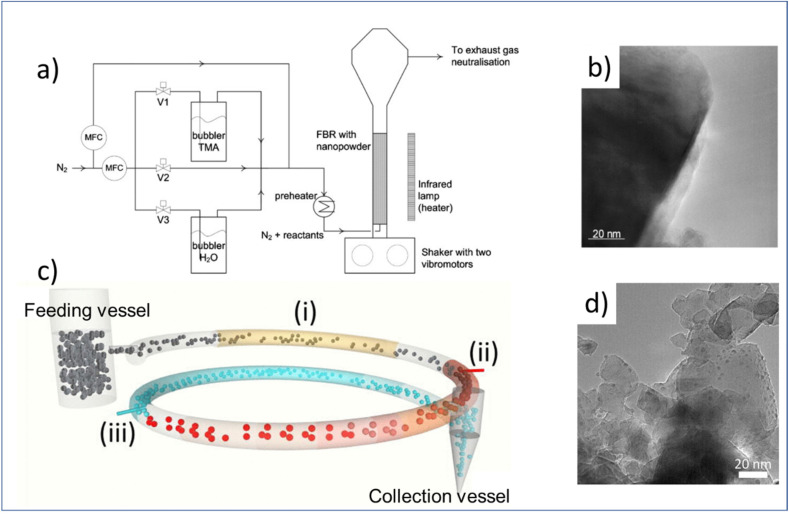
Atmospheric-pressure ALD on high-porosity particles. (a) fluidized bed ALD reactor to particle coating, (b) TEM image of a particle obtained by fluidized bed ALD, (c) schematic of the s-ALD reactor consisting of a fluidized feeding vessel, a pneumatic transport line made of three segments: preheating (i), precursor reaction zone (ii), co-reactant reaction zone (iii), and a collection vessel, (d) TEM image of Pt/TiO_2_ samples obtained by s-ALD. Reprinted with permission from Beetstra *et al.*^119^ and Van Ommen *et al.*^121^

To make fluidized-bed ALD more economically scalable, one can consider operation under atmospheric pressure for the coating of particles. This has received increased attention in recent years. Beetstra *et al.*,^119^ for the first time, tested atmospheric-pressure ALD of Al_2_O_3_ in a fluidized bed reactor to coat LiMn_2_O_4_ particles (200–500 nm) for battery applications, ranging from 5 to 28 cycles (Fig. 9a and b). The resulting coatings were characterized by transmission electron microscopy (TEM), energy dispersive X-ray (EDX) spectroscopy, scanning electron microscope (SEM) and X-ray diffraction (XRD). The results showed that the individual particles were coated homogeneously. Later on, Soria-Hoyo *et al.*^122^ demonstrated the potential of a scalable fluidized bed ALD reactor design for the production of a stable CO_2_ sorbent by coating CaO on a nano-silica powder matrix. The CO_2_ capture capacity of the coated materials was tested in a few cycles of thermogravimetric analysis (TGA). The results indicated that the synthesized materials were more stable than the limestone-derived CaO. In the same year, the group proposed a novel s-ALD reactor design consisting of a fluidized feeding vessel, a pneumatic transport line and a collection vessel, allowing the continuous production of nanoparticles at atmospheric pressure (Fig. 9c and d).^121^ More recently, the same group employed the fluidized bed ALD reactor to prepare photocatalytic core–shell samples with tunable activity *via* depositing an ultrathin layer of SiO_2_ on TiO_2_ nanoparticles.^126^ The deposition process was carried out at a temperature as low as 100 °C with silicon tetrachloride (SiCl_4_) as the precursor and H_2_O as the co-reactant. Surprisingly, a substantially lower chlorine impurity was observed in the deposited SiO_2_ layer which could hardly be achieved by low-pressure ALD.^138,139^ The photocatalytic property of the obtained TiO_2_/SiO_2_ nanostructures was proven by the degradation experiments with Rhodamine B (RhB) solution. The results suggested that the highest photocatalytic activity of the particles was achieved at a SiO_2_ layer thickness of 0.7 nm. Conversely, the performance of the photocatalyst was strongly suppressed when the SiO_2_ layer was thicker than 1.4 nm.

Another important application of fluidized-bed ALD is in the pharmaceutical domain. The ability to obtain pharmaceutical particles with tailor-made size, shape and surface properties has significant implications for drug delivery and therapeutic applications. Zhang *et al.*^128,129^ demonstrated the successful, complete and conformal layering of Al_2_O_3_ films on drug particles in a fluidized ALD reactor at near-atmospheric pressure. With a few ALD cycles, the properties of drug particles such as their dissolution, dispersibility and heat transfer can be improved and this way the release and utilization of ALD-modified drug particles can be further optimized. Also, La Zara *et al.*^130^ compared the effect of different coatings, including ALD-grown Al_2_O_3_, TiO_2_, SiO_2_, and MLD-grown PET and titanicone, on the wettability of drug particles. The ceramic ALD films were most effective in improving the hydrophilicity of the drugs while PET films, made by molecular layer deposition (MLD) – the organic counterpart of ALD, were effective in delivering hydrophobic powders.

Overall, potential applications of nanoparticles developed by fluidized-bed atmospheric-pressure ALD in various areas are listed in literature (Table 3). Developing fluidized bed reactors for ALD at an industrial scale is expected to be viable and in addition, to be much more convenient under atmospheric pressure.^137^ However, one can also expect that high-humidity air flows entering the reactor, may lead to unwanted side reactions. This is especially important when the second precursor being used is water.

**Table 3:** Overview of atmospheric-pressure ALD in new emerging application fields

Materials	Precursor	Co-reactant	Substrate	Deposition temperature	Applications	ALD type	Year	Ref.
Al_2_O_3_	TMA	H_2_O	Particles	160 °C	Li-ion batteries	t-ALD	2009	119 a
Pt	MeCpPtMe_3_	O_3_	Particles	250 °C	Catalysis	t-ALD	2013	120
Pt	MeCpPtMe_3_	O_2_	Particles	100/250 °C	Catalysis	s-ALD	2015	121 a
CaO	Ca(thd)_2_	O_3_	Particles	250 °C	CO_2_ capture	t-ALD	2015	122
Al_2_O_3_	TMA	H_2_O	Particles	27 ± 3 °C	—	t-ALD	2015	123
Pt	MeCpPtMe_3_	Air	Graphene	100 °C	Catalysis	t-ALD	2017	124
Cu_2_O	Cu(i)(hfac)	H_2_O	Particles	250 °C	Photocatalysis	t-ALD	2021	125
SiO_2_	SiCl_4_	H_2_O	Particles	100 °C	Photocatalysis	t-ALD	2020	126
PET	TC	EG	Particles	150 °C	Photoactivity	MLD	2020	127
Al_2_O_3_	TMA	H_2_O	Particles	100 °C	Drug delivery	t-ALD	2017	128
Al_2_O_3_	TMA	O_3_	Particles	30 °C	Drug delivery	t-ALD	2019	129
Al_2_O_3_	TMA	O_3_	Particles	40 °C	Drug delivery	t-ALD	2021	130
TiO_2_	TiCl_4_	H_2_O	Particles	40 °C	Drug delivery	t-ALD	2021	130
SiO_2_	SiCl_4_	H_2_O	Particles	40 °C	Drug delivery	t-ALD	2021	130
PET	TC	EG	Particles	150 °C	Drug delivery	MLD	2021	130
Titanicone	TiCl_4_	EG	Particles	120 °C	Drug delivery	MLD	2021	130
Al_2_O_3_	TMA	H_2_O	Particles	180–300 °C	—	t-ALD	2021	131
Al_2_O_3_	TMA	H_2_O	Capillary column	300 °C	GC	t-ALD	2022	16
TiO_2_	TiCl_4_	H_2_O	Membrane	180 °C	Water treatment	t-ALD	2017	132
ZnO	DEZ	H_2_O	Membrane	RT	Separation	s-ALD	2022	133
Al_2_O_3_	TMA	H_2_O	Cotton fabric	100 °C	—	t-ALD	2011	14

a See also Fig. 9 for illustrations.

A few commercial companies working on the coating of nanoparticles with atmospheric-pressure ALD are worthwhile mentioning. One start-up company, Delft IMP, is dedicated to commercializing its ALD and MLD technologies for depositing ultrathin layers on powder surfaces, particularly for energy transition applications like batteries, catalysts, fuel cells and electrolysers.^140^

On a parallel track, Forge Nano has developed and commercialized a continuous vibrating bed ALD reactor named ‘CIRCE’.^141^ This reactor is designed for s-ALD mode mass production at atmospheric pressure with a high production capacity up to 4000 kg h^−1^ and >99% product yield. The reactor can be used at temperatures ranging from 50 to 200 °C, making it suitable for various kinds of particles and coating materials.

#### 3.2.2. Capillary columns for gas chromatography

**Fig. 10:**
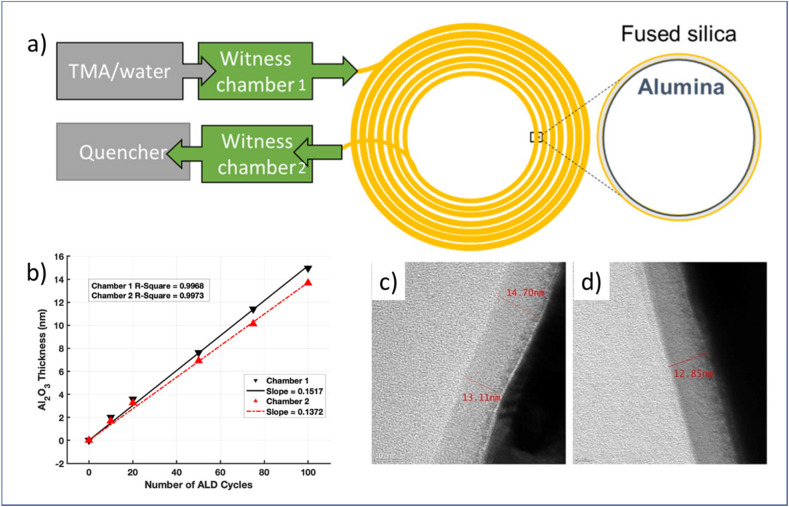
(a) Schematic overview of flow-through atmospheric t-ALD for the deposition of Al_2_O_3_ on the capillary column, (b) measured Al_2_O_3_ thickness grown on silicon in two different chambers after different numbers of ALD cycles, and TEM images of the respective film thickness at the entrance (c) and end (d) of the capillary column after 100 ALD cycles. From Patel *et al.*^16^ Copyright 2022, American Chemical Society.

Capillary columns are one of the most important components of gas chromatography (GC), an indispensable tool in analytical chemistry. Modification and functionalization by coating the internal surface of a capillary column are key in tuning these columns for fast response analysis and long lifetime. Typically, a GC capillary column has an inner diameter of ∼0.1–0.5 mm and a length of 15–60 m.^16^ This special structure makes it quite challenging to deposit thin films on the inner surface of the column with high uniformity. To address this issue, Patel *et al.*^16^ designed a flow-through atmospheric-pressure ALD reactor that allows for the coating of Al_2_O_3_ on long (5–12 m), narrow bore (0.53 mm) capillaries. As shown in Fig. 10, two witness chambers with silicon witness samples inside were placed at the inlet side and exit side of the capillary. The film thickness and composition on silicon samples were then analyzed by spectroscopic ellipsometry (SE) and X-ray photoelectron spectroscopy (XPS), respectively. The GPC values measured on the witness samples in the two chambers were 1.5 and 1.4 Å per cycle, respectively, showing a slight difference in film thickness. In addition, the thicknesses of the Al_2_O_3_ film at the entrance (13.1–14.7 nm) and at its end (12.9 nm) of the 5 m long capillary were close to each other, as measured by TEM (Fig. 10c and d). Results on the 12 m long capillary were similar, showing the reproducibility potential of ALD deposition on a complicated structure at near-atmospheric pressure.

#### 3.2.3. Membrane modification

Membrane technology has been widely used in various industries, for applications such as water treatment, gas separation and chemical separation. Efficient separations rely on well-defined pore sizes and surface chemistry of the membranes used. Membrane modification enables a more efficient separation *via* precise tuning of the pore size and/or surface chemistry to realize higher selectivity and/or improved antifouling ability. Among the various modification methods, ALD is considered to be a promising new route for producing membranes with well-controlled characteristics at the nanoscale, due to its precise control of both the chemistry and physical nature of the membrane pore surface. Only recently, the modification of membranes by ALD has been extensively studied by researchers.^142,143^ In particular, the use of ALD/MLD technologies to tune the selectivity between the di-valent and mono-valent ions has shown great potential for separation purposes in nanofiltration (NF) and reverse osmosis (RO) membranes.^144–146^ Also, ALD-enabled catalytic membranes are expected to improve the chemical conversion efficiency and to alleviate the membrane fouling issues in water treatment.^147–149^ In addition, ALD/MLD modified membranes can enhance the selectivity between the small molecules in gas separation membranes *via* defect curing and/or pore narrowing.^150,151^ For more information, the reader is referred to recent reviews, which have comprehensively discussed the benefits of ALD in their applications in various aspects of membranes.^142,143^ However, the majority of studies thus far used conventional low-pressure ALD for the modification of membranes on planar sheets or disc-shaped substrates. It is extra challenging to prepare membranes with a more complicated structure such as hollow fiber geometry or multichannel geometry, which are more widely used in practice. In addition, at vacuum or low-pressure conditions, it is far less economically viable to upscale the technology for large-area production of membranes.

**Fig. 11:**
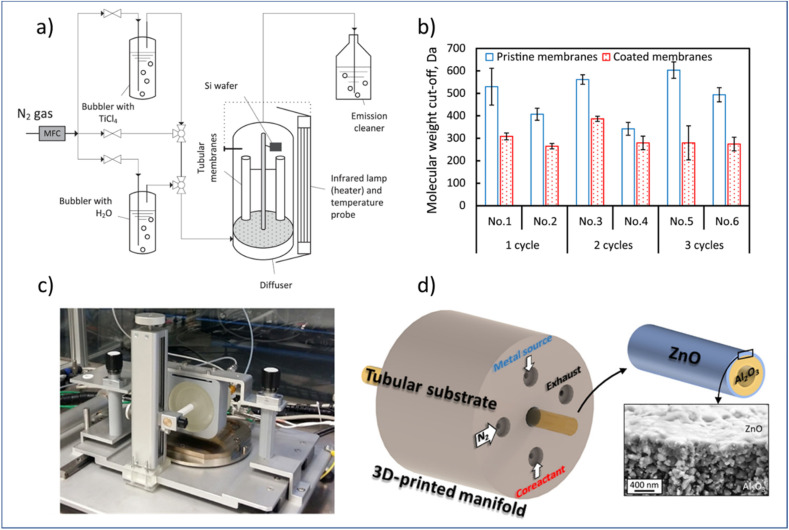
Atmospheric-pressure ALD for membrane modification. (a) Schematic overview of a t-ALD setup, (b) MWCO of membranes before and after ALD modification; (c) photograph of an atmospheric s-ALD setup, and (d) configuration of its injection head for the growth of ZnO on the outer surface of a ceramic Al_2_O_3_ tubular membrane, and cross-section micrograph of the grown ZnO film. From Shang *et al.*^132^ and Toldra-Reig *et al.*^133^ (Copyright 2022, American Chemical Society).

In this regard, researchers have studied the potential of using both atmospheric-pressure t-ALD and s-ALD for membrane fabrication and modification. For example, Shang *et al.*^132^ prepared tight ceramic NF membranes by a flow-type atmospheric-pressure t-ALD reactor (Fig. 11a). Two commercial ceramic NF tubes were vertically placed in an up-flow reactor and a silicon wafer was fixed as a reference sample next to the membrane to monitor the thickness of the coated layer. The molecular weight cut-off (MWCO) of the membranes was reduced to a range of 260 to 380 Da from an initial value of 450 Da after one to three cycles of TiO_2_ coating from TiCl_4_ and water vapor. However, a high water permeability (11–16 L m^−2^ h^−1^ bar^−1^) of these modified ceramic membranes was maintained, which is notably higher than commercial polymeric membranes and sol–gel-made ceramic NF membranes with a similar MWCO (∼300 Da). The growth rate of the layer was found to be much smaller than the one deposited at the planar silicon surface, which could be ascribed to the steric hindrance of the TiCl_4_ molecules into the pores of NF membranes. In another study, Toldra-Reig *et al.*^133^ explored the deposition of thin ZnO films on tubular ceramic membranes by designing and fabricating a customized 3D-printed s-ALD manifold (Fig. 11c). To ensure a homogeneous deposition on the membrane surface, the geometry of the gas manifold was optimized by CFD simulation prior to design. The deposition of the ZnO layer with such a gas manifold was validated on both a tubular Cu foil and a porous Al_2_O_3_ tubular membrane. The results indicated that s-ALD is a promising route for high-rate deposition of high-quality conformal thin films on complex substrates. However, due to a major CVD contribution, the GPC of the ZnO layer was measured to be 4 Å per cycle, which is much higher than that of the conventional ALD. In addition, the membrane properties such as pore size and water permeance were not yet investigated in this study. Furthermore, the manifold design used only allows for the deposition of ALD layers on the outer surface of the membrane, making it hard to be compatible with commercial multichannel ceramic membranes.

## 4. Conformality of atmospheric-pressure ALD on porous substrates

In an ALD deposition process, uniform coating on planar substrates is mainly determined by the chemisorption and subsequent chemical reactions of the precursors and co-reactants. In such a reaction-limited domain, the effect of operating pressures on the uniformity of the layer on a planar surface is not a limiting factor. Therefore, working at a higher (atmospheric) pressure could simplify the reactor design and make this technology affordable for users in the capital extensive (*i.e.*, non-semiconductor) industry. However, for highly porous materials, the deposition of conformal coatings is much more challenging at higher pressures than at vacuum conditions due to the diffusion limitations in the (meso)pores. In this section, we will first discuss gas transport in porous materials at various pressures and pore size scales. Next, the factors determining the conformal coating on porous substrates by atmospheric-pressure ALD will be assessed. Finally, we provide our perspectives on the development of a dedicated reactor design to improve the conformality of coatings on a porous substrate at atmospheric pressure.

### 4.1. Gas transport

Theoretically, the transport of gases can be divided into two main regimes: viscous flow (molecular diffusion) and molecular flow (Knudsen diffusion).^152^ To distinguish the difference between these two flow regimes, the Knudsen number *K*_n_ (a dimensionless parameter) is introduced, and defined by the mean free path *λ* (m) and the pore diameter *d*_p_ (m) as:1
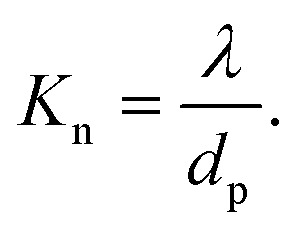


The mean free path *λ* of molecules is mainly affected by three key factors: temperature *T* (K), molecule size *d* (m) and pressure *p* (Pa). To describe their relationships, the following equation is given:2
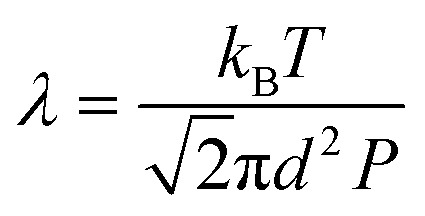
where *k*_B_ is the Boltzmann constant.

**Fig. 12:**
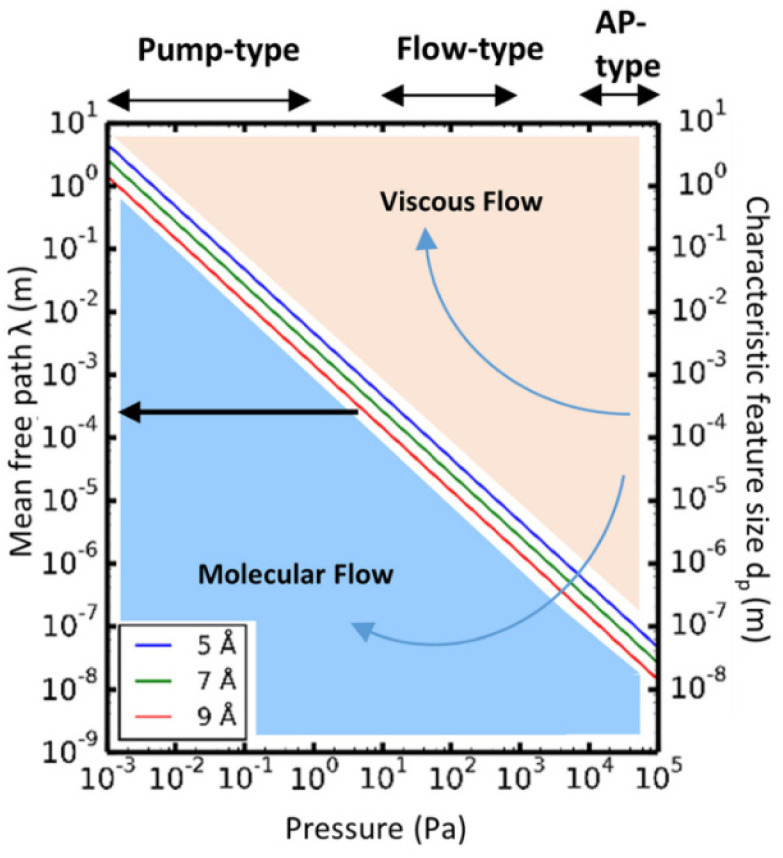
Mean free path (left *y*-axis) as a function of pressure, calculated according to eqn (2), for molecules with average diameters of 5, 7, and 9 Å, respectively, at a temperature of 100 °C. The working pressure regimes of the pump-type, flow-type, and atmospheric pressure (AP-type) ALD reactors are indicated in the figure. The right *y*-axis of the graphs shows the characteristic feature size (*d*_p_). Comparing *d*_p_ with the mean free path, *λ*, allows determination of the corresponding flow regime for a given pressure: molecular flow regime (*λ* ≫ *d*_p_) and viscous flow regime (*λ* ≪ *d*_p_). From Cremers *et al.*^152^

Given the above theoretical equations, two flow regimes can be separated based on the mean free path, the pore diameter of the substrate and the pressure of the system, as shown in Fig. 12. In the *viscous flow regime* (*K*_n_ ≪ 1), the mean free path of the molecules is much smaller than the pore diameter of the substrates. In this case, inter-particle interactions dominate the transport process. On the contrary, in the *molecular flow regime* (*K*_n_ ≫ 1) the mean free path of the molecules is much larger than the pore diameter of the substrates, leading to particle–surface interactions as the main transport phenomenon. Therefore, in very low pressure condition (pump-type reactor), molecular flow can be easily achieved even in macroscopic structures. Thus, in a conventional temporal ALD reactor, molecular flow can often be obtained on porous substrates with macropores. Whereas, at near-atmospheric pressures, the molecular flow will only be realized in nanopore structures.^152^

### 4.2. ALD conformality at atmospheric pressure

The conformality of layers grown with ALD inside porous substrates is determined by three key parameters: the reaction probability, the pore aspect ratio, and the precursor diffusion coefficient.^135^ We can assume the reaction probability to be the same for the internal and external parts of the porous substrate systems. In this way, the saturation dose of ALD is defined at the diffusion-limited regime, where the diffusion coefficient is extremely important. From Fig. 12, the transition from viscous flow to molecular flow is shown for the change in pore size of the substrate and for the change of reactor pressures. To analytically describe the effect of the reactor pressure and the pore size on the diffusion coefficient, an effective diffusion coefficient *D*_eff_ was thus proposed by Poodt *et al.*,^153^ and defined as3
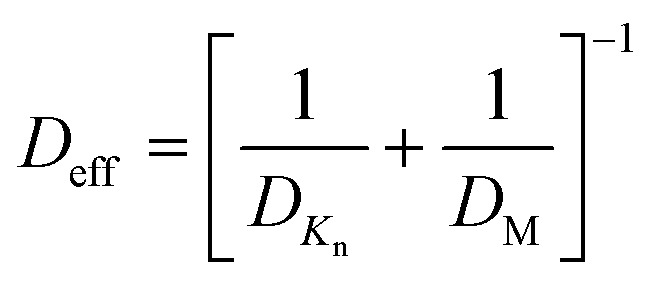
where *D*_*K*_n__ is the Knudsen diffusion coefficient, and *D*_M_ is the molecular diffusion coefficient.

**Fig. 13:**
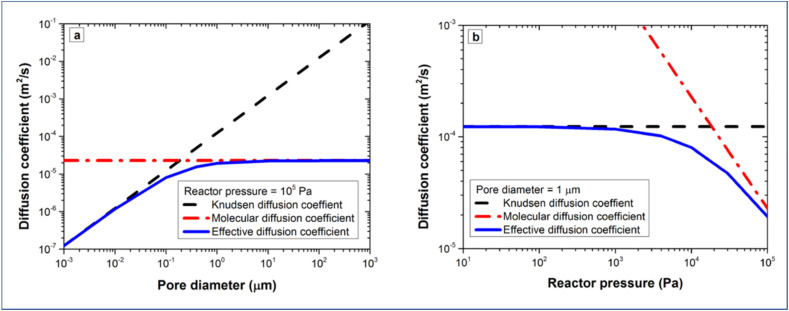
Diffusion coefficients of TMA as a function of (a) pore size at a fixed pressure of 10^5^ Pa, and (b) reactor pressure for a fixed pore size of 1 μm. From Poodt *et al.*^153^

The molecular diffusion coefficient (*D*_M_) is only pressure dependent, thus irrelevant to the pore size of the substrate. In other words, the diffusion coefficient is increased with decreasing pressure, and *vice versa*. On the contrary, the Knudsen diffusion coefficient (*D*_*K*_n__) is dependent on the pore size of the substrate and independent of the overall pressure. With these three parameters (the molecular, Knudsen, and effective diffusion coefficients), one can plot these for a precursor either as a function of pore size at atmospheric pressure or as a function of reactor pressure for a certain pore size (Fig. 13).

If we take TMA as an example, it can be seen that Knudsen diffusion still dominates when the pore size of the substrate is smaller than 0.5 μm at atmospheric pressure (Fig. 13a). For a 1 μm diameter pore, Knudsen diffusion prevails at a reactor pressure of up to 10^4^ Pa (Fig. 13b).

**Fig. 14:**
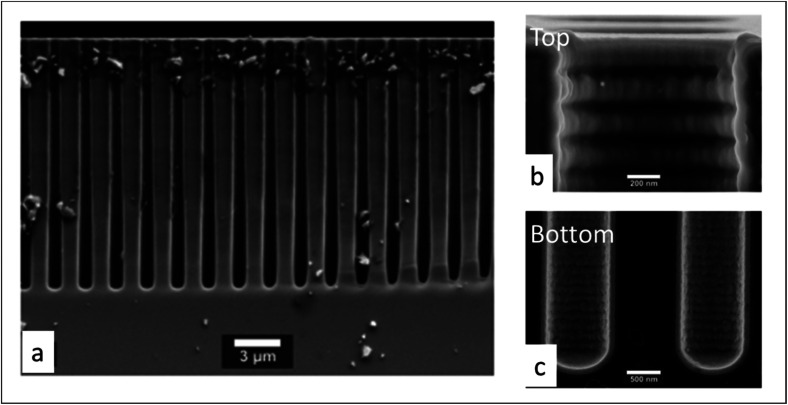
Cross-sectional SEM images of 1 μm diameter and 17 μm deep pores array in silicon, conformally coated by atmospheric ALD of Al_2_O_3_. (a) Global structure, (b) pore opening and (c) pore bottom. From Poodt *et al.*^153^

Next, the saturation dose of a precursor required to completely cover the walls of a pore can be estimated by including the pressure-dependent diffusion coefficient (*D*_eff_) in the model of Gordon *et al.*^154^ Exposure of a substrate to a certain gas dose is determined by the product of its partial pressure and the exposure time. Therefore, for a given pore structure with known pore size and pore length, the saturation dose and saturation time can be mathematically calculated for different pressures. In their study Poodt *et al.*^153^ found that, for a 1 μm diameter and 50 μm deep circular pore, the saturation time could be 10 times less at atmospheric pressure as compared to a low-pressure condition (133 Pa), although the saturation dose could be 2 times higher. In atmospheric-pressure ALD, a much higher precursor partial pressure is allowed, and thus shorter saturation times can be achieved than for low-pressure ALD. To further demonstrate the feasibility of atmospheric-pressure ALD for conformal coating on substrates with macropores, Poodt *et al.*^153^ coated a silicon wafer substrate with 17 μm deep pores of 1 μm diameter created by a Bosch-type Deep Reactive Ion Etching (DRIE) process. To make sure that a near-to-complete step coverage was achieved, a long precursor exposure time (∼250 ms) per cycle was used (*cf.* 10 ms for planar substrates). As shown in Fig. 14, for such a high aspect ratio structure, conformal coating (100% step coverage) was successfully realized with s-ALD at atmospheric pressure despite that molecular diffusion dominates in this pore dimension.

**Fig. 15:**
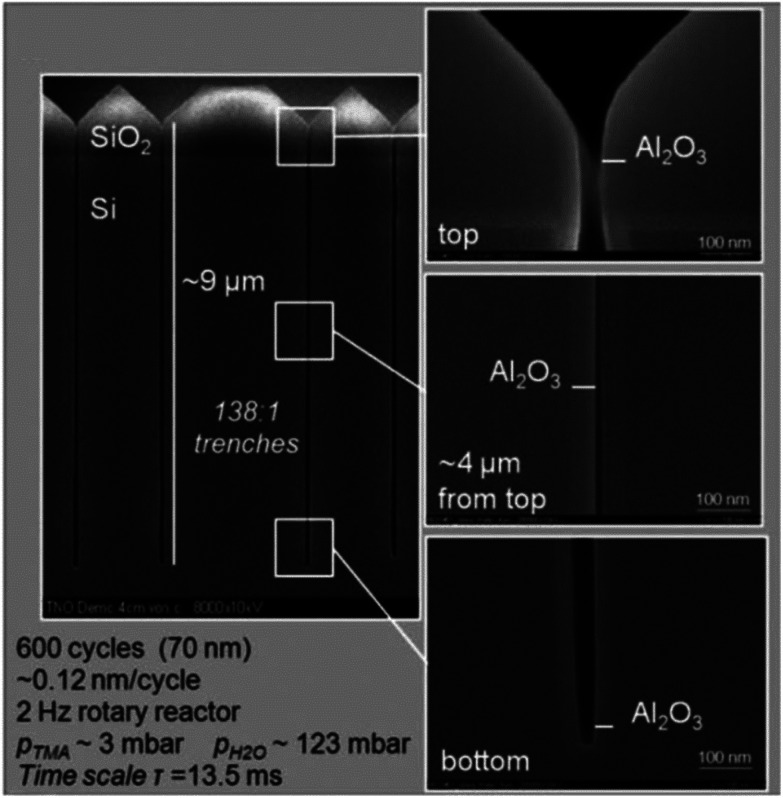
Cross-sectional SEM images of an Al_2_O_3_ layer deposited at 1 atm. and 200 °C in 138 : 1 aspect ratio trenches during 600 cycles in a rotary s-ALD reactor. (Trenched wafers kindly provided by Fraunhofer CNT/Namlab, Dresden). From Roozeboom *et al.*^155^

The conformal coating of thin films on high aspect ratio substrates at atmospheric pressure was also confirmed by Roozeboom *et al.*^155^ In their study, arrays of trenches with an aspect ratio as high as 138 : 1 were used for the deposition of an Al_2_O_3_ layer from TMA and H_2_O. In such a high aspect ratio structure with 65 nm trench openings and 9 μm trench depth, the conformal coating was realized at 1 atm. and 200 °C in a rotatory s-ALD reactor with a cycle time of 13.5 ms (Fig. 15).

Overall, for small pore diameters, Knudsen diffusion dominates the transport of molecules, even for atmospheric-pressure ALD. As a result, the saturation dose is the same for both low- and high reactor pressure. Whereas, in terms of large pore diameters, higher saturation doses are required for high-pressure reactors than for low-pressure reactors. Benefiting from the high precursor partial pressure, the saturation time of the precursor is even shorter for atmospheric ALD than for low-pressure ALD.

### 4.3. Dedicated reactor design

Despite the fact that conformal coating in atmospheric-pressure ALD is confirmed to be feasible on porous substrates theoretically and experimentally, most studies report on substrates with regular geometry (*e.g.* Si-based trench arrays and anodic aluminum oxide (AAO)) to simplify the modelling or to facilitate substrate characterization after coating.^152^ In reality, far more complex pore geometries and substrate porosity are expected. To study the coating effect on those substrates, a dedicated reactor design is highly desired.

George and co-workers were one of the first to develop an ALD reactor system which allows for *in situ* monitoring of pore size variation of a tubular Al_2_O_3_ membrane with the number of ALD cycles.^156,157^ The initial pore size of the membrane was 50 Å and the pore diameter reduction was monitored using *in situ* N_2_ and Ar permeation measurements. By assuming Knudsen diffusion and using an aperture pore model, the pore size of the membrane can then be estimated by permeation results. The authors investigated the Al_2_O_3_ membrane coating with ALD Al_2_O_3_, SiO_2_, and TiO_2_, and found the pore diameter reduction rates to vary among these materials. This could be partially explained by the different deposition temperatures and thus the different hydroxyl surface group concentrations present on the material surface. In addition, the final pore diameters may reach a minimum value which is defined by the molecular size of the reactant or the molecular size of the gases used for permeation measurements. These results demonstrated the potential of ALD in tailoring nanopores of membranes for specific applications.

**Fig. 16:**
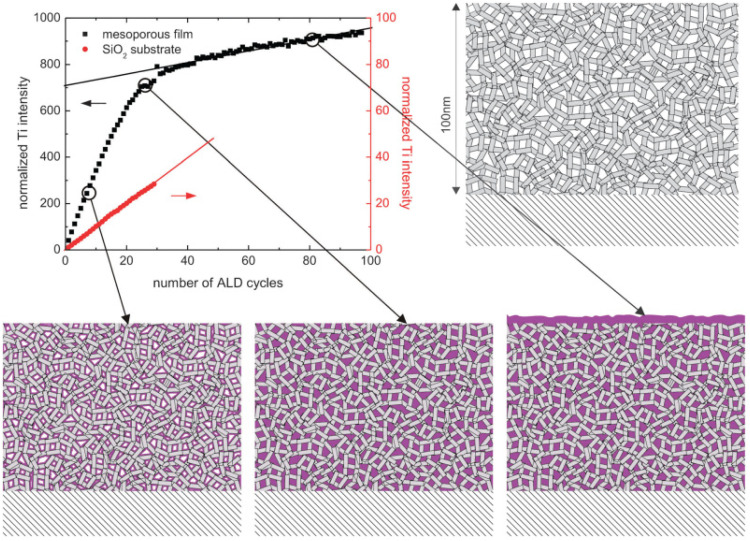
Normalized Ti XRF intensity as a function of the number of TiO_2_ ALD cycles on a planar SiO_2_/Si substrate and on a mesoporous substrate with an initial average pore size of 4 nm. The cartoons display the gradual pore filling until completion followed by surface growth only. From Dendooven *et al.*^161^

With *in situ* gas permeation measurements, however, only the information on pore size variations can be obtained. To gain more information (*e.g.*, porosity and pore size distribution) about the penetration of ALD coatings into substrates with mesopores and/or nanopores, Dendooven *et al.*^158–162^ developed several approaches by implementing *in situ* characterization into an ALD reactor. As shown in Fig. 16, *in situ* X-ray fluorescence (XRF) was employed to monitor the Ti uptake during the deposition of TiO_2_ in mesoporous films with an initial average pore size of 4 nm as well as on a planar SiO_2_ surface.^161,162^ The Ti XRF intensity from the mesoporous substrate increased much faster than the one deposited on a planar SiO_2_ substrate in the first few cycles, suggesting the penetration and deposition of an ALD layer inside the mesopores. With the increase of ALD cycles, the intensity increase of the Ti-signal from the mesoporous film slowed down due to the narrowing down of pore width as well as the decrease of accessible interior surface area. After a certain number of cycles, the slope of the Ti-intensity curve became constant, indicating that the deposition continued only on top of the outer surface as the pores were completely filled with TiO_2_. Also, the ALD reactor could be integrated with other *in situ* characterization methods such as ellipsometric porosimetry (EP)^160^ and grazing incidence small-angle X-ray scattering (GISAXS).^158^ This way, EP could provide information on the porosity and pore size distribution during the ALD growth on mesoporous or nanoporous features, and with GISAXS one could monitor the evolution in density and internal surface area as growth progresses. Especially with the combination of such advanced *in situ* characterization techniques, better insights on the pore-filling mechanism can be obtained.^158,159^

Currently, *in situ* characterization techniques of ALD layers grown on porous substrates are typically available in low-pressure ALD reactors only. However, some techniques can be expected to be more easily integrated into atmospheric-pressure ALD reactors. For example, the *in situ* N_2_ and Ar permeation measurements are easier to be achieved since no vacuum system is required.

### 4.4. Challenges and opportunities

**Fig. 17:**
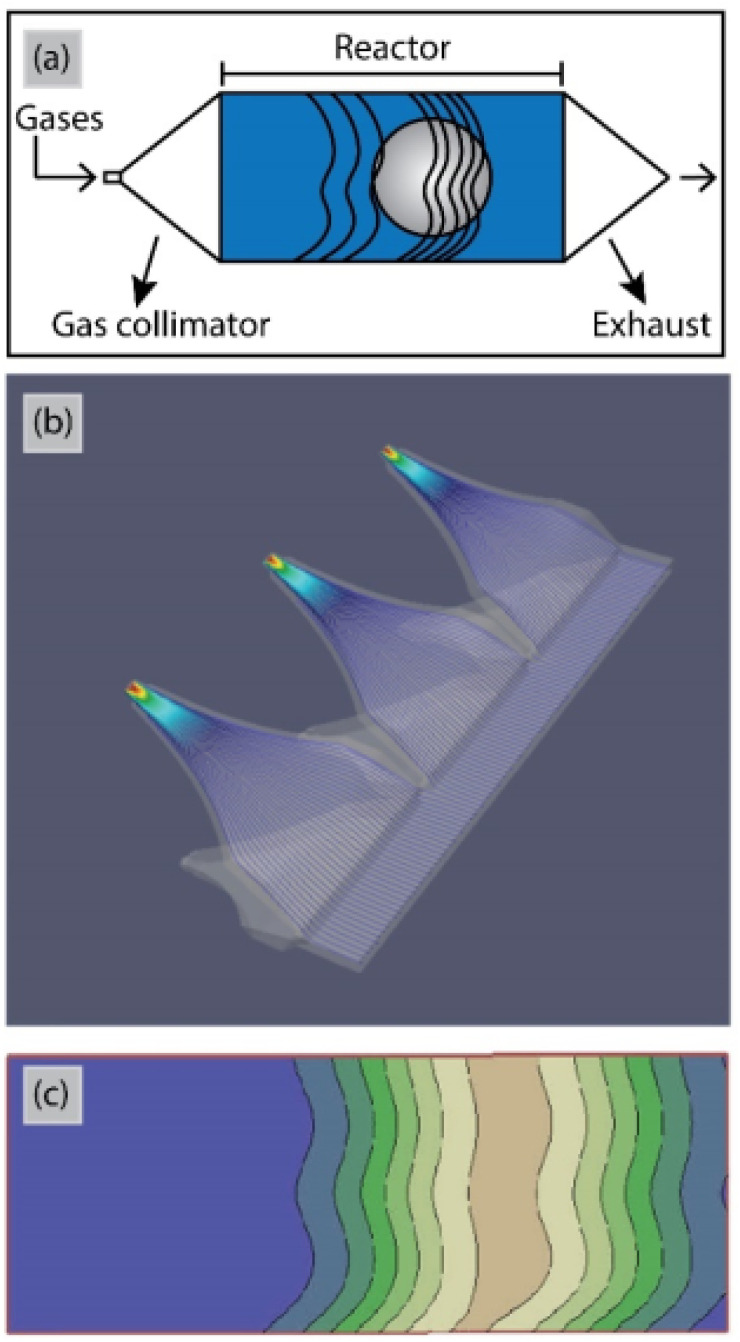
(a) Schematic top view and (b) computational flow dynamics simulation of the gas injectors in the Encapsulix parallel precursor wave (PPW) system. (c) Iso-contour lines for precursor pulsed wave fronts after propagation of the precursor pulses in the reaction space.^163^

So far for porous materials and/or 3D substrates, the majority of ALD reactors has been used in temporal mode (Table 3). The main challenge for working at atmospheric pressure with t-ALD is the long purging times due to the low diffusion rate of gases. Increasing the inert gas velocity during purging steps can shorten the purging times. According to a study by Mousa *et al.*,^15^ the gas velocity for t-ALD in atmospheric mode had to be increased by >350 times to maintain the same purging time as that in conventional low-pressure mode. A unique concept for high deposition rates is the semi-s-ALD (or spatio-temporal) reactor developed by Encapsulix,^163^ as illustrated in Fig. 17. In this reactor design, a gas collimator which provides parallel precursor waves is used as a gas injector. In this approach, a constant laminar flow of nitrogen is supplied through the reactor while a sequence of millisecond-long pulses of extremely collimated precursor flows is injected. The gas confinement into wave fronts ensures the separation of reactants and exposure of a single gas or gas mixture to the stationary substrates at a time. With such a design, high throughput deposition of encapsulation layers for OLED displays has been realized. However, the reactor is used under vacuum conditions, thus further exploration with computational flow dynamics simulation, *etc*. is needed to assess options for operation at atmospheric pressure.

Most of the reactor design and process aspects described above can be traced back, amongst others, to the early and fundamental work published by Giling,^164^ who investigated gas flow patterns in several horizontal reactor designs by interference holography. In general, the flow patterns and their stability while propagating from the entrance to the outlet of a reactor are mainly impacted by three factors: (1) the occurrence of turbulence due to (too) high gas flow rates, (2) the occurrence of thermal convection as a result of temperature gradients along the reactor height axis which can disturb laminar flow regime, and (3) any reactor entrance and outlet effects causing undeveloped flow and temperature profiles. For example, Giling reported that the thermal entrance length should often be several times longer than the flow entrance length. These thermal entrance effects should be taken into account in developing new reactor designs, in terms of optimized inlet length, adding a preheating zone and optimizing the free height of a reactor. In occasional cases, carrier gases with a high thermal capacitance, like H_2_ and He are easier in establishing stable laminar flow compared to N_2_ and Ar.^164^ However, they are less cost-effective.

## 5. Conclusions

The intrinsic advantages of atomic-scale thickness control, coating uniformity and 3D conformality of functional layers have made ALD the preferred technology of choice in the field of ultrathin film deposition. This preference is no longer restricted to applications in semiconductor manufacturing, where ALD is essential to continue the scaling of nanoelectronic devices, but also in new emerging fields. This review provides a comprehensive analysis and information from the point of view of new application, with our focus being on the niche of atmospheric-pressure ALD, and new applications in the tuning and functionalization of high-porosity materials (powders, membranes for nanofiltration, *etc*.).

Each application requires a specific reactor design optimized for its intended purpose. For example, large-area/foil substrates (*e.g.*, solar cells and displays), require a R2R design, while single-wafer ALD is suitable for rigid wafers. Powder coating necessitates a fluidized-bed design, whereas flow-through or shower head designs are preferable for other applications. The design of each concept is tailored to meet the specific demands of the electronics industry and the unique characteristics of different substrates, such as their porosity.

In addition, considerations on flow dynamics at high *vs.* low operation pressure and challenges and opportunities associated with the reactor design are included, as well as some recent highlights in patterned deposition and Molecular Layer Deposition.

## Author contributions

M. Chen: literature collection, resources, writing – original draft, review & editing. M. P. Nijboer: writing – review & editing. A. Y. Kovalgin: writing – review & editing. A. Nijmeijer: writing – review & editing. F. Roozeboom: conceptualization, writing – review & editing. M. W. J. Luiten-Olieman: conceptualization, writing – review & editing, funding acquisition.

## Conflicts of interest

There are no conflicts to declare.

## Supplementary Material
